# A fluorescence lifetime-based novel method for accurate lipid quantification of BODIPY vital-stained *C. elegans*

**DOI:** 10.1016/j.jlr.2024.100646

**Published:** 2024-09-19

**Authors:** Chen Xu, Jintao Luo, Yong Yu

**Affiliations:** State Key Laboratory of Cellular Stress Biology, School of Life Sciences, Faculty of Medicine and Life Sciences, Xiamen University, Xiamen, China

**Keywords:** lipid droplets, lipids, fluorescence microscopy, fatty acid/transport, triglycerides, BODIPY, *C. elegans*, fat quantification, fluorescence lifetime, lysosome-related organelles

## Abstract

Lipid droplets (LDs) are organelles associated with lipid storage and energy metabolism, thus, their morphology and quantity are of significant research interest. While commercially available BODIPY dye effectively labels LDs in various cell types, it also labels lysosome-related organelles (LROs) in *C*. *elegans*, leading to non-specific LD quantification. Here, we report that the fluorescent signals of BODIPY exhibit distinct fluorescence lifetime patterns for LROs and LDs, which can be captured, visualized, and filtered by fluorescence lifetime imaging microscopy. Furthermore, we proposed and validated a method based on fluorescence lifetime that can improve the accuracy of fat storage quantification in BODIPY vital-staining worms, which holds broad applications, including rapid and accurate LD quantification in forward genetic screening. Additionally, our method enables observing dynamic LD-LRO interactions in living worms, a unique capability of BODIPY vital staining. Our findings highlight distinct BODIPY fluorescence lifetime characteristics of LDs and LROs, providing a valuable tool for future research on LDs, LROs, or their interactions.

As obesity has become a significant global public health issue, an in-depth investigation into fat storage is imperative (1). Lipid droplets (LDs), the primary organelles for fat storage, have garnered increasing interest. They store triacylglycerols and cholesterol esters and are evolutionarily conserved in most eukaryotic and prokaryotic cells (2, 3, 4). Recent evidence suggests that LDs are not merely fat storage sites but also play crucial roles in lipid metabolism. LDs interact with cellular compartments such as the endoplasmic reticulum and lysosomes to maintain lipid metabolic balance (5, 6). Moreover, the quantity and size of LDs influence lifespan (7, 8). Research on LDs may also provide novel insights into treating diseases such as diabetes and non-alcoholic fatty liver disease (9, 10, 11). Therefore, studying LDs is extremely important. *Caenorhabditis elegans*, with its complete genome sequence, ease of genetic screening, and conservation of energy regulation pathways with humans, is an excellent model organism for LD research (12, 13).

As a permeable fluorescent dye, BODIPY stains LDs effectively with considerable fluorescence quantum yield, making it widely used in cellular LD studies (14). However, in *C. elegans*, BODIPY vital-staining shows low accuracy in fat storage quantification. Research has shown that, besides LDs, BODIPY also stains lysosome-related organelles (LROs) (15, 16). LROs are gut granules with spontaneous fluorescence but are not fat-storage organelles in *C. elegans* (16, 17). Both off-target staining and spontaneous fluorescence of LROs result in unreliable signals that interfere with the detection of LDs and the accuracy of fat storage quantification in live worms.

Fluorescence lifetime imaging microscopy offers a novel approach, combining traditional fluorescence intensity imaging with time-resolved fluorescence information (18). Fluorescence lifetime is the average time a fluorophore remains excited in a specific environment. It is a material constantly influenced by local chemical environments such as pH or polarity, rather than dye concentration (19, 20). Internal environments of LDs and LROs are distinct—LDs primarily store neutral lipids, while LROs have an acidic and polar internal environment (15)—such differences provide possibilities of distinguishable BODIPY fluorescent lifetimes.

In this study, based on fluorescence lifetimes, we distinguished LROs and LDs stained with BODIPY. Additionally, through a series of parameter analyses of staining effects, we developed an effective method termed fluorescence lifetime filter (FLF). FLF reduces the interference of BODIPY from LROs in fat storage quantification, increasing the accuracy of fat storage measurements. Due to its live vital-staining capability, operational convenience, and high accuracy in detecting LDs under fluorescence lifetime filtering (FLF), BODIPY is expected to be an efficient and powerful tool for research on forward genetic screening for fat storage, morphological phenotypes of LDs and LROs, as well as for observing their dynamic behaviors in worms.

## Materials and methods

### *C. elegans* strains and maintenance

The WT strain used in this study was N2, and the following mutants were also employed: *glo-1*(*zu391*), *daf-22*(*ok693*), and *daf-2*(*e1370*), all obtained from *Caenorhabditis* Genetics Center. We also used *raxIs118[sur-5p:ctns-1::RFP-3XHA;unc-76(+)]* (21) and *ldrIs [mdt-28p::mdt-28::mCherry; unc-76(+)]* (22) transgenic strains. Unless otherwise specified, all the *C. elegans* strains were maintained and propagated at 20°C on NGM (Nematode Growth Medium) plates with *E. coli* OP50 using standard procedures (23).

### BODIPY and Lysotracker Red live staining

BODIPY 493/503 (catalog number D3922) and Lysotracker Red DND-99 (catalog number L7528) were purchased from Thermo Fisher Scientific and dissolved in DMSO to a stock concentration of 2 mM, stored at −20°C. The stock solution was diluted in PBS to prepare staining solutions of the required concentrations. All strains were imaged at the day-1-adult stage. Twenty-four hours before reaching this stage, 400 μl of the diluted staining solution was added to 6 cm diameter NGM plates containing worms. For BODIPY and LR co-staining, a mixed dye solution containing them was added, each at a final concentration of 1 μM in a total volume of 400 μl. The plates were gently shaken to evenly distribute the solution, dried on a laminar flow clean bench in the dark, and then continued to culture in the dark. When the worms reached the state for imaging, they were washed off NGM plates with M9T (M9 + 0.2% Tween), washed three times with M9T, and then anesthetized with 20 mM levamisole for 10 min. Subsequently, the worms were placed on 2% agarose pads and then imaged under the Leica STELLARIS 8 STED microscope system.

### Fixed Nile Red staining

Nile Red was dissolved in DMSO to a stock concentration of 0.5 mg/ml and stored at −20°C. The stock solution was diluted in 40% isopropanol. Day 1 adult worms were washed off NGM plates with M9T, washed twice with M9T, fixed in 2% Paraformaldehyde on a shaker for 30 min, and frozen at −80°C for at least 2 h. Subsequently, worms were thawed, washed twice with M9T, dehydrated in 40% isopropanol for 3 min, stained with the 1.65 μg/ml, 300 μl diluted staining solution for 30 min in the dark, washed twice with M9T, and immediately imaged under a fluorescent microscope.

### Tau contrast imaging

Tau contrast imaging was performed using a Leica STELLARIS 8 STED microscope system with a white laser as the light source, delivering 85% of maximum power output. Tau contrast images were obtained using the BODIPY channel in Tau contrast mode. BODIPY channel in Tau contrast imaging utilized an excitation wavelength of 494 nm and an emission range of 499 nm–713 nm. When the BODIPY channel simultaneously images with LR intensity, it utilizes an excitation wavelength of 502 nm and an emission range of 507–572 nm. Imaging resolutions were set at 1024 × 1024 (using 10×, 20×, and 63× objectives) or 512 × 512 (using a 40× objective for spontaneous fluorescence imaging).

### Fluorescence intensity imaging and FLF

Unfiltered fluorescence intensity imaging and FLF of BODIPY stained worms were performed using the same microscope system, excitation and absorption bands, and imaging resolutions set with Tau contrast imaging. Unfiltered fluorescence intensity was obtained by using the maximum lifetime gate (−1∼11.5 ns) in Tau gating mode, while FLF used 4∼11.5 ns.

LR/mCherry/RFP fluorescence intensity imaging was performed using the same microscope system and imaging resolutions set with Tau contrast imaging. The excitation wavelengths were set at 573 nm and the emission wavelength range was 578–750 nm.

Fluorescence intensity of fixed Nile Red Imaging was performed using a Zeiss Axioplan2 imaging microscope with a 20x objective, AxioCam MRm camera, and Nile Red filter. Appropriate exposure parameters were used to avoid overexposure, and they were kept constant for experimental and control groups within the same batch.

### Time-lapse imaging

XYZT mode was employed using Tau mode, capturing images with a 63× objective. Four layers, each 0.99 μm thick, were imaged for each sample, and maximum brightness projection was applied. Images were captured every 3.8 s for a total of 30 frames. Image resolution was set at 512 × 512 pixels. Maximum brightness projection was applied to the four-layer images collected in the Tau contrast channel for each time point in ImageJ.

### Calculation and statistic

Fluorescence loss rate was calculated as:

(1) $$Fluorescence\;loss\;rate=\left(1-\frac{\left\langle I_{\;f}\right\rangle }{\left\langle I_{uf}\right\rangle }\right)\times 100\%$$

where $\left\langle I_{\;f}\right\rangle$ is the mean fluorescence intensity of LDs or LROs after filtered by fluorescence lifetime gate, $\left\langle I_{\;uf}\right\rangle$ is the unfiltered mean fluorescence intensity of LDs or LROs.

RLL was quantified as the ratio of the average intensity of selected LDs and LROs.

(2) $$RLL=\frac{\left\langle I_{LDs}\right\rangle }{\left\langle I_{LROs}\right\rangle }$$

where $\left\langle I_{LDs}\right\rangle$ is the mean fluorescence intensity of LDs, $\left\langle I_{LROs}\right\rangle$ is the mean fluorescence intensity of LROs.

Every normalization data were normalized to the corresponding control group.

(3) $$I_{\left\langle normalized\right\rangle }=\frac{I_{\left\langle unnormalized\right\rangle }}{I_{\left\langle control,average\right\rangle }}$$

where $I_{\left\langle normalized\right\rangle }$ is the mean fluorescence intensity of control or experimental groups after normalized, $I_{\left\langle unnormalized\right\rangle }$ is the mean fluorescence intensity of control or experimental groups before normalized, $I_{\left\langle control,average\right\rangle }$ is the average data of mean fluorescence intensity of the control group.

## Results

### Worms vital stained with BODIPY exhibited distinct fluorescence lifetimes for LDs and LROs

**Fig. 1:**
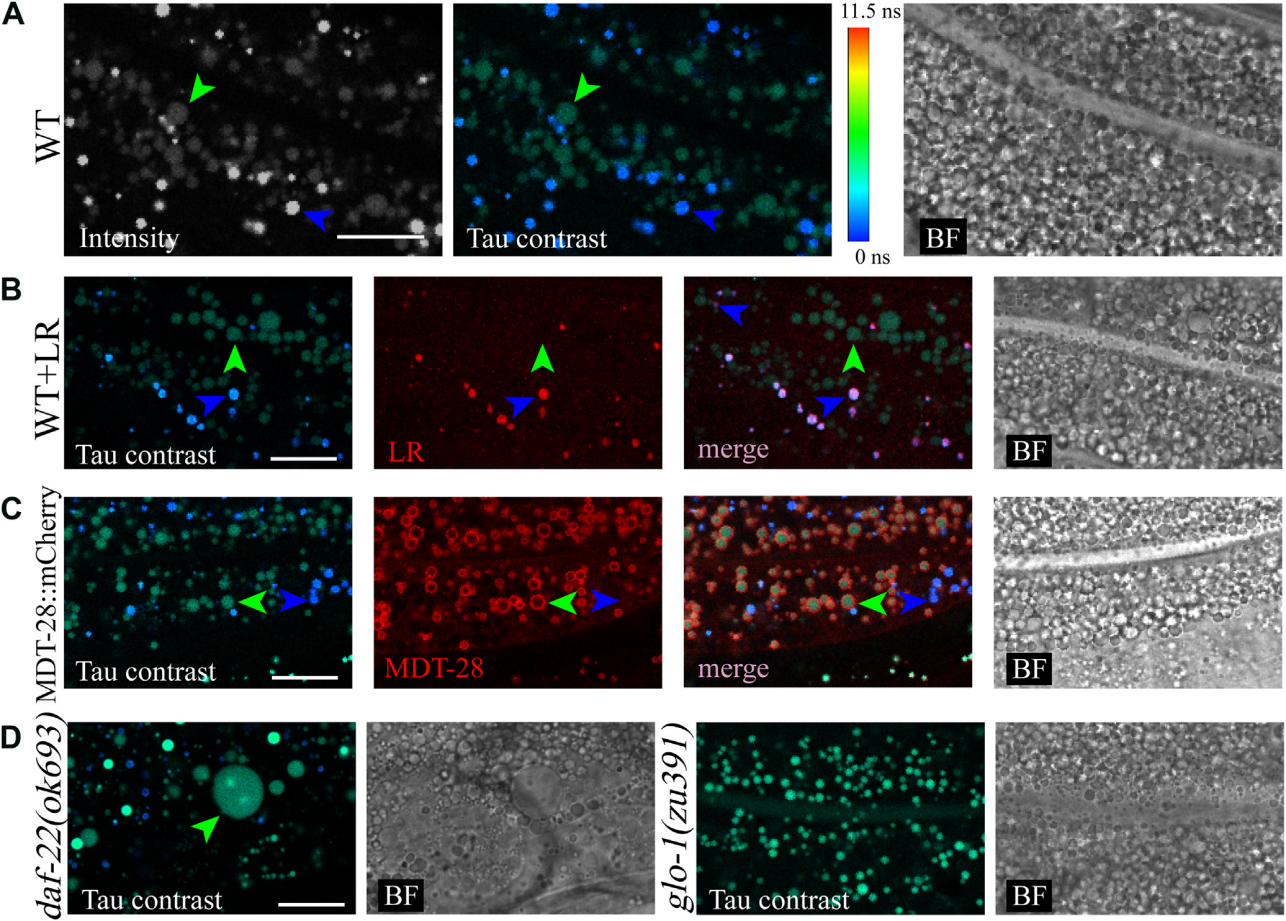
BODIPY vital-stains LDs and LROs with distinct fluorescence lifetimes. A: WT worms were live-stained with 1 μM BODIPY. The fluorescence intensity image, obtained in the manner of unfiltered fluorescence intensity, revealed regions with varying fluorescence intensities in the intestines of the worms. The Tau contrast image displayed two regions with distinct fluorescence lifetimes, characterized by color variation. The color bar represents the relationship between color and fluorescence lifetime in all Tau contrast images. B: WT worms were live co-stained with a mixed dye solution containing BODIPY and LR. Regions with short fluorescence lifetime in the Tau contrast image (blue arrow) overlapped with red regions in the LR channel, while regions with long fluorescence lifetime did not (green arrow). C: MDT-28::mCherry transgenic worms were live-stained with 1 μM BODIPY. LDs marked by red circles in the MDT-28 channel overlapped with regions of long fluorescence lifetime (green arrow) in the Tau contrast images. D: The *daf-22(ok693)* mutant and the *glo-1(zu391)* mutant were separately live-stained with 1 μM BODIPY. The *daf-22(ok693)* mutant exhibited large LDs (green arrow), while the *glo-1(zu391)* mutant, lacking LROs, showed no blue regions in the Tau contrast image. Scale bar = 10 μm. WT, wild-type; LR, Lysotracker Red DND-99; LDs, lipid droplets; LROs, lysosome-related organelles; BF, bright field.

To acquire fluorescence lifetime images, we vital-stained wild-type (WT) *C. elegans* by feeding them BODIPY and we simultaneously imaged fluorescence intensity and Tau contrast. In Tau contrast images, differences in fluorescence intensity were represented by brightness, while distinctions in fluorescence lifetimes were indicated by different colors shown with the fluorescence lifetime color bar (Fig. 1A). This color bar was applied to all Tau contrast images in this study. We observed two regions in the intestine with distinct fluorescence lifetimes, appearing green and blue. Additionally, areas with short fluorescence lifetimes exhibited high fluorescence intensity (blue arrow), whereas areas with long fluorescence lifetimes showed low fluorescence (green arrow).

To further identify the cellular organelles associated with different fluorescence lifetimes, we conducted multiple colocalization experiments. First, we used Lysotracker Red DND-99 (LR), a commercial lysosomal dye that stains acidic LROs within the worms’ intestines (15).

We co-fed LR and BODIPY to WT worms and performed Tau contrast imaging and LR fluorescence intensity imaging simultaneously (Fig. 1B). In merge images, we found that the blue regions in the Tau contrast image overlapped with red regions in the LR channel, whereas the green regions did not. This indicated that LR specifically accumulates in regions with low fluorescence lifetimes in Tau contrast images.

Additionally, we used CTNS-1::RFP as a lysosomal marker. CTNS-1 is a lysosomal cystine transporter located on the lysosome membrane (24), used to label lysosomes in *C. elegans* (25, 26, 27). We stained *raxIs118[sur-5p:ctns-1::RFP-3XHA;unc-76(+)]* worms with BODIPY and performed Tau contrast imaging and RFP fluorescence intensity imaging simultaneously (Supplemental Fig. S1). We observed that RFP overlapped with the blue regions in Tau contrast images, suggesting that the blue regions in Tau contrast images may represent lysosomes. These results indicated that regions with short fluorescence lifetimes in BODIPY vital-stained worms are LROs (lysosomes).

Subsequently, we used MDT-28 fused with mCherry, an LD membrane protein (22, 28) as an LD marker. We stained *ldrIs[mdt-28p::mdt-28::mCherry;unc-76(+)]* worms with BODIPY and performed Tau contrast imaging and mCherry fluorescence intensity imaging simultaneously (Fig. 1C). The red circles in the mCherry channel overlapped with the green regions in Tau contrast images but not with the blue regions, indicating that regions with long fluorescence lifetimes in BODIPY vital-stained worms are LDs.

Furthermore, we examined the fluorescence lifetime of BODIPY in LD and LRO-defective mutants. Previous studies have shown that *daf-22(ok693)* mutants possess enlarged LDs (29), while *glo-1(zu391)* mutants lack LROs almost entirely (30).

Consistent with these findings, Tau contrast images showed a significantly large green region in *daf-22(ok693)* mutants (Fig. 1D), whereas no blue regions were observed in *glo-1(zu391)* mutants (Fig. 1E). This demonstrated the capability of Tau contrast imaging to accurately characterize LDs and LROs with morphological and numerical mutations.

In summary, our results demonstrated that LDs and LROs exhibit distinct fluorescence lifetimes in worms vital-stained with BODIPY. The BODIPY fluorescence signals originating from LDs displayed long lifetimes, while those from LROs displayed short lifetimes.

### Dye concentrations and fluorescence lifetime filters influence the fluorescence intensity gap between LDs and LROs under BODIPY vital staining

**Fig. 2:**
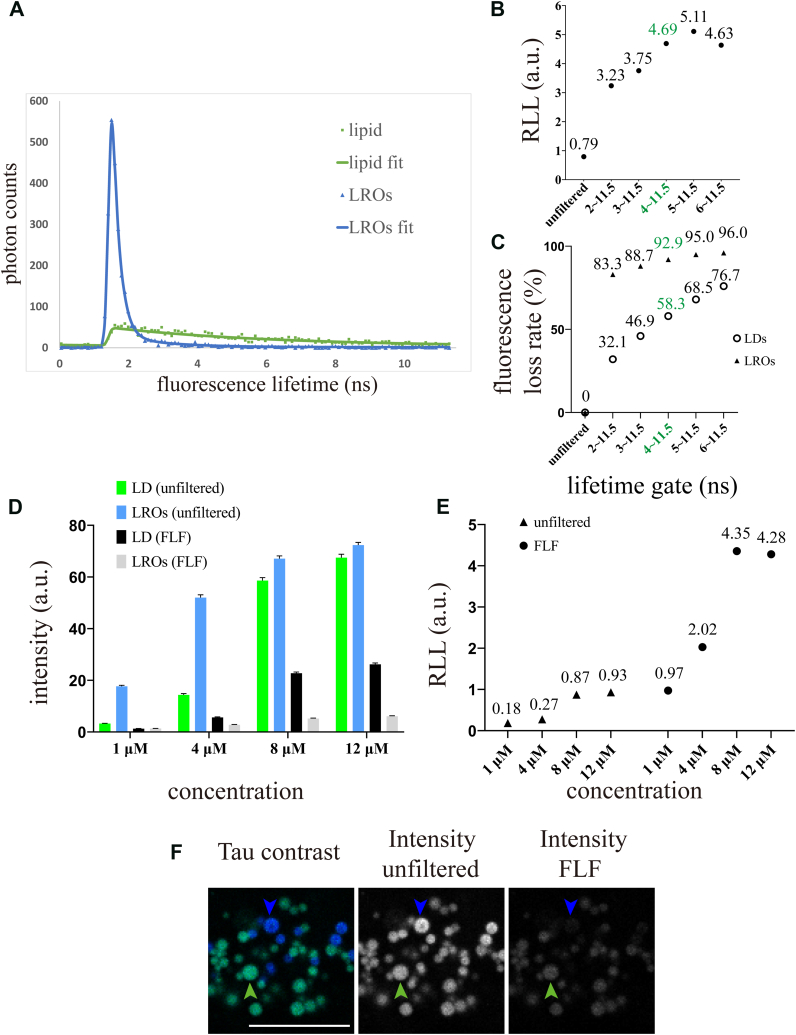
Using 8 μM BODIPY for staining and a fluorescence lifetime filter significantly filters out LROs fluorescence. A: WT worms were live-stained with 8 μM BODIPY. The fluorescence lifetime distribution analysis was performed in LAS X software. Scatter plot data and fitted curves indicate different fluorescence lifetime distributions for the selected pair of LDs and LROs. For a good fit, n-Exponential Reconvolution fit model was selected, the Exponential Components value for each curve was 2, and the χ^2^ value ranged between 0.5 and 1.2. B: The same LD and LRO pair from (A) was imaged using various lifetime gates set in Tau gating mode. Their fluorescence intensities were measured, and the RLL was calculated. C: The same LD and LRO pair from (A) was imaged with different lifetime gates set in Tau gating mode, and the fluorescence loss rates of LDs and LROs were calculated. The green highlight in (B) and (C) indicates the optimal fluorescence lifetime gate we selected. D: WT worms were live-stained with varying concentrations of BODIPY (1, 4, 8, and 12 μM). The mean fluorescence intensity of selected LDs and LROs was measured. Fluorescence lifetime filtering attenuated the fluorescence signal of LROs more than that of LDs. The data was shown as mean ± SEM (n = 50 LDs or LROs per column). The 50 samples were obtained from five worms, with the ten brightest LDs or LROs selected per worm. E: The RLL was calculated using the data from (D), increasing with higher concentration BODIPY and the use of FLF. F: WT worms were live-stained with 8 μM BODIPY. The figures show the fluorescence intensity of LDs and LROs with or without FLF. Scale bar = 10 μm. a.u. = arbitrary unit. RLL, the fluorescence intensity ratio of LDs to LROs; FLF, fluorescence lifetime filter.

To quantify the fluorescence signal from LDs, we aimed to reduce the fluorescence signal from LROs. We imaged a pair of intestinal LROs and LDs at optimal focal planes using FLIM imaging mode on the Leica STELLARIS 8 STED. This pair of intestinal LROs and LDs was selected as regions of interest (ROI) to measure the fluorescence lifetime distribution using LAS X software. As shown in Fig. 2A, most photons from LROs exhibited a narrow lifetime distribution, peaking at 1.5 ns, while the photons from LDs showed a much broader lifetime distribution. By setting appropriate fluorescence lifetime gates, only photons with specific lifetimes were selectively collected, thus achieving fluorescence intensity filtering based on fluorescence lifetime (Supplemental Fig. S2). We implemented this filtering through Tau gating mode and sought to determine the optimal time gate range. Since almost no LRO fluorescence signals had lifetimes exceeding 4 ns, long-pass fluorescence lifetime gates were suitable for eliminating LRO signals. Thus, the maximum limit of the gate was set to the maximum value in Tau gating mode, 11.5 ns.

We sequentially applied various long-pass fluorescence lifetime gates with different minimum thresholds in Tau gating mode and imaged pairs of LDs and LROs. We found that adjusting the minimum thresholds had two main effects: (1) it altered the interference of LRO fluorescence signals with LDs, and (2) it impacted LD brightness. To quantify LRO interference on LDs, we measured the mean fluorescence intensity of LDs and LROs using ImageJ and calculated the fluorescence intensity ratio of LDs to LROs (RLL). A higher RLL indicates a lower relative fluorescence intensity of LROs compared to LDs. To assess LD brightness, we determined the fluorescence loss rate caused by filtering. The RLL and fluorescence loss rates for LDs and LROs under different Tau gating settings are shown in Fig. 2B and C. We observed that, compared to unfiltered data, RLL increased with higher minimum thresholds in the lifetime gate. However, this improvement in RLL came at the cost of increased fluorescence loss for LDs, indicating reduced LD brightness. Notably, the fluorescence loss rate for LROs was much higher than that for LDs, providing a basis for distinguishing between the two. Furthermore, the enhanced RLL reduced the relative fluorescence intensity of LROs, thereby improving the accuracy of LD quantification, though it also diminished the overall LD signal. By balancing LD quantification accuracy with signal preservation, we determined that the optimal fluorescence lifetime gate was between 4 and 11.5 ns. Under this setting, the fluorescence loss rate for LDs was 58.3%, while that for LROs was 92.9%. We termed this method the Fluorescence Lifetime Filter (FLF).

Next, we evaluated the effects of FLF under different BODIPY concentrations. We measured the fluorescence intensities of numerous LDs and LROs from multiple worms, with and without FLF (Fig. 2D). As BODIPY concentration increased, the fluorescence intensity of LDs increased proportionally more than that of LROs, suggesting that high BODIPY concentrations can enhance the fluorescence intensity of intestinal LDs and reduce the intensity gap between LDs and LROs. The results showed that FLF efficiently filtered LRO signals while preserving LD signals, especially at high BODIPY concentrations. With FLF, the preserved fluorescence intensity of LDs was much higher than that of LROs. Calculations based on fluorescence intensity data revealed that both FLF and high concentrations increase RLL (Fig. 2E). Notably, the combination of the two methods exhibited an additive effect. Using 8 μM BODIPY with FLF, the RLL reached a peak of 4.35 (Fig. 2E), allowing for efficient visualization of LDs without interference from LROs in *C. elegans* (Fig. 2F).

Previous studies indicated that LROs exhibit spontaneous fluorescence that increases with age (17, 27). Therefore, spontaneous fluorescence in aging individuals may affect the accuracy of fat storage quantification. We imaged unstained WT worms of different ages in the BODIPY channel using Tau contrast mode and found that spontaneous fluorescence consistently had short lifetimes, regardless of the worms' age and fluorescence intensity (Supplemental Fig. S3). Since FLF is based on specific fluorescence lifetime distributions, it filters out all signals outside the gate, irrespective of their source. Hence, we anticipate that FLF can also eliminate age-related spontaneous fluorescence from LROs, enabling accurate characterization of LDs in older worms. In summary, the FLF method combined with 8 μM BODIPY staining provides reliable LD signals.

### High dye concentration and fluorescence lifetime filter enable accurate fat quantification under BODIPY vital-staining

**Fig. 3:**
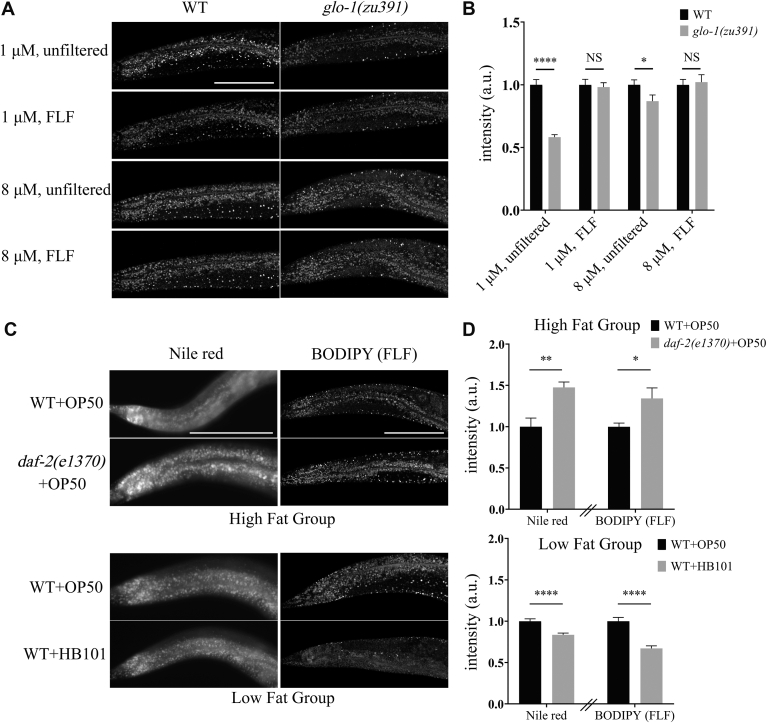
FLF and 8 μM BODIPY showed convincing fat quantification results. A: WT worms and *glo-1(zu391)* mutants were live-stained with 1 or 8 μM BODIPY. The fluorescence intensity of the two types of worms in the same experiment condition can be compared with each other, but not between experimental conditions. Scale bar = 100 μm. B: The fat storage of the two types of worms in (A) are quantified by fluorescence intensity. The statistical graph shows that both high concentration and FLF can enhance the accuracy of fat quantification of WT and *glo-1(zu391)* mutants. Data of *glo-1(zu391)* were normalized to the corresponding WT group. (mean ± SEM; n ≥ 19/column; ∗∗∗∗*P* < 0.0001; ∗*P* < 0.05; Data were analyzed with an unpaired *t* test.) C: WT + HB101 has a low-fat phenotype, *daf-2(e1370)* + OP50 has a high-fat phenotype, and WT + OP50 is the control for both of them. Worms in the BODIPY (FLF) group were live-stained with 8 μM BODIPY, while worms in the Nile red group were fix-stained with Nile red. These images show the fluorescence intensity of worms with different fat phenotypes under two staining methods. Fluorescence intensity can be compared between the same staining methods in the same fat group. Scale bar = 100 μm. D: The fat storage of the two groups in (C) are quantified by fluorescence intensity. The fat quantification results in worms with high or low-fat phenotypes of both methods show similar trends. The results quantified by the two methods are separated by “//”, we are not inclined to directly compare the data from the two methods. High or low fat group was normalized to their respective control group. (mean ± SEM; n ≥ 13/column; ∗∗∗∗*P* < 0.0001; ∗∗*P* < 0.01; ∗*P* < 0.05; Data was analyzed with an unpaired *t* test.) a.u. = arbitrary unit.

To verify the improvement of fat quantification accuracy with high BODIPY concentration and FLF, we compared fat storage between WT worms and *glo-1(zu391)* mutants under various experimental conditions (Fig. 3A). In our study, fat storage was quantified using mean fluorescence intensity, and the intestinal region between the tail and the last egg was delineated as the ROI. Previous research has demonstrated that *glo-1* mutants show no difference in fat storage compared to WT worms (31).

We found that the 1 μM BODIPY without FLF group showed a significant difference in fluorescence intensity between WT and *glo-1(zu391)* mutants. Both FLF and increased dye concentration reduced the fluorescence intensity gap between the two strains. The statistical differences between them under different experimental conditions negatively correlated with RLL (Figs. 3A and B and 2E), demonstrating that RLL reflects the accuracy of fat storage quantification.

However, the combination of FLF and 8 μM BODIPY, which showed higher RLL (Fig. 2E), did not further improve the accuracy of fat quantification in this case. We speculate that this is due to the semi-quantitative nature of the method used, where even a modest increase in RLL can significantly improve accuracy. Nonetheless, achieving higher RLL is still crucial, especially when more precise quantitative methods are required.

Next, we quantified fat storage in worms with different fat storage phenotypes using 8 μM BODIPY and the FLF method (Fig. 3C). We compared the results with another well-established worm’s fat storage quantification method: Nile Red fixed staining (32, 33, 34). We utilized three groups for the experiment: *daf-2(e1370)* mutants [a strain with a high-fat phenotype (35)] fed with OP50, WT worms fed with HB101 bacteria (which results in relatively lower fat accumulation compared to OP50 bacteria (36)) and WT worms fed with OP50 as the control group for both comparisons. Additionally, the Nile Red fixed staining groups were cultured simultaneously and received an equal volume of DMSO diluted with PBS as a control. The statistical results indicated that our method showed a similar trend in quantifying the two lipid phenotype groups compared to Nile Red fixed staining method, demonstrating that our method is feasible for fat quantification (Fig. 3D). Thus, we concluded that staining with 8 μM BODIPY and using FLF allows for accurate quantification of fat storage in live *C. elegans*.

### Applications of fluorescence lifetime imaging in BODIPY vital-staining worms

**Fig. 4:**
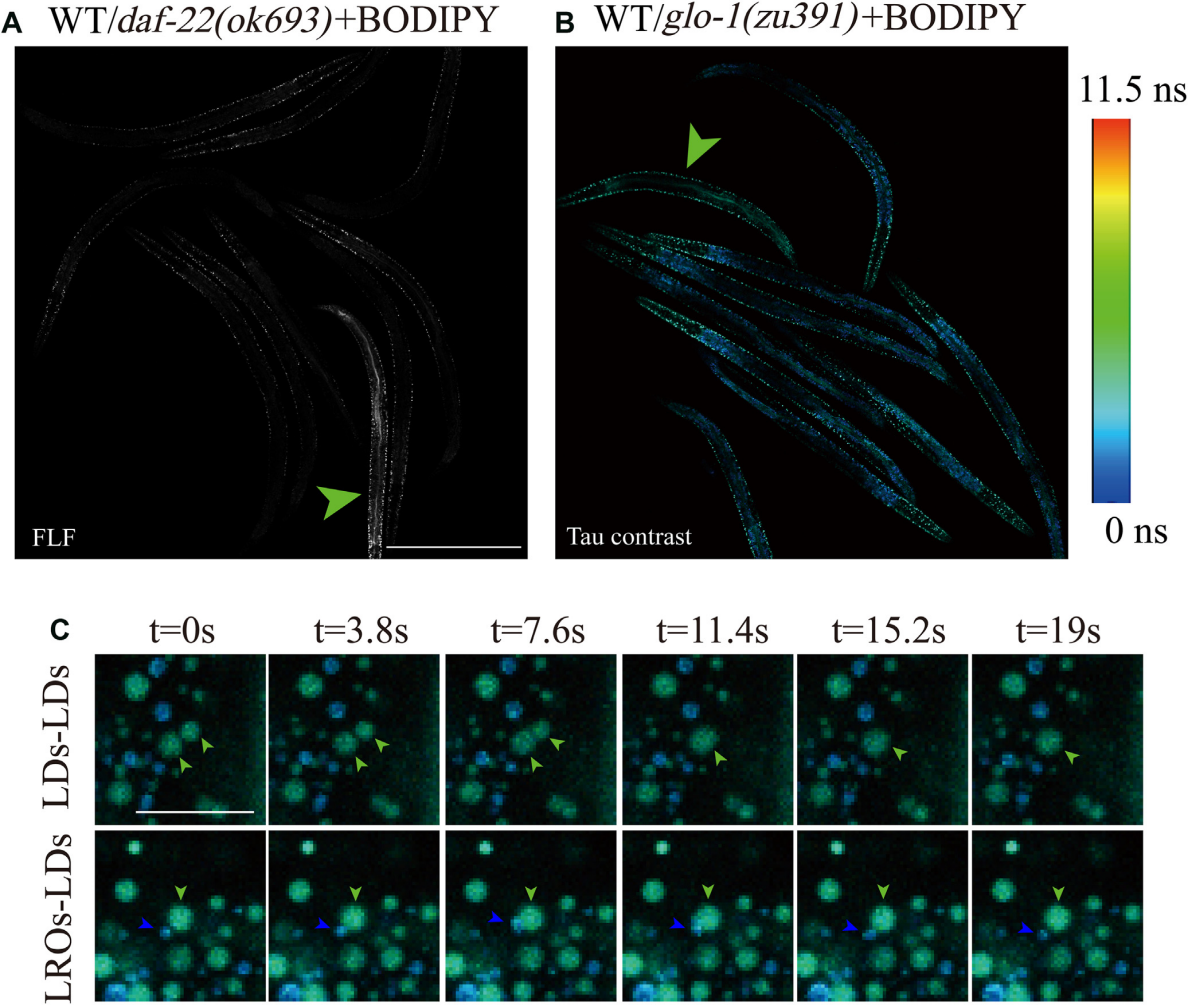
The Tau contrast images and the FLF method present potential applications in living imaging. A and B: Nine WT worms and one mutant were mixed and live-stained with 8 μM BODIPY. Worms with abnormal phenotypes of LDs (A) or LROs (B) can be clearly observed in FLF or Tau contrast images under a 10x objective lens (green arrows). Scale bar = 300 μm. C: *daf-22(ok693)* mutants were live-stained with 8 μM BODIPY. The dynamic behavior of LDs and LROs was observed in anesthetized worms. The LDs-LDs image showed two smaller LDs merging into a larger LD within 19 s (green arrows). The LROs-LDs image showed an LRO (blue arrow) and an LD (green arrow) interacting in a kiss-and-release manner within 19 s. Scale bar = 5 μm.

Due to the advantages of live staining, BODIPY is expected to be used for forward genetic screening for fat storage after improving the accuracy of worm fat quantification through our method. Additionally, the distinction between LROs and LDs based on fluorescence lifetime makes the screening for LRO mutants feasible. We conducted a simulation experiment to demonstrate the potential feasibility of the FLF method in forward genetic screening. Nine WT worms and one mutant stained with 8 μM BODIPY were mixed at the L4 stage. They were transferred to the center of a confocal dish, washed with M9, anesthetized with 20 mM levamisole for ten minutes, and imaged using FLF or Tau contrast with a 10x objective lens. We found that it was easy to quickly identify the *daf-22(ok693)* mutant with large LDs from the images after FLF (Fig. 4A) and to identify the *glo-1(zu391)* mutant with an abnormal LRO phenotype in the Tau contrast image (Fig. 4B). After imaging, the identified mutants were isolated, recovered with M9. Worms typically regained full activity within 12 h after recovering from anesthesia and they were further cultured on fresh NGM plates. After the worms laid eggs, genotyping was conducted. Our experiments demonstrate that 8 μM BODIPY combined with FLF (or Tau contrast) can clearly display mutants with abnormal LDs (or LROs) under low magnification, enabling high-throughput screening. Additionally, our experimental process is convenient and fast, with low damage to worms, demonstrating the feasibility of using BODIPY for forward genetic screening for fat storage and LD and LRO morphology.

Furthermore, the characteristics of BODIPY live staining for LDs and LROs can also be utilized to conveniently study the dynamics of LDs and LROs simultaneously in Tau contrast images. Using time-lapse imaging, we imaged BODIPY live-stained *daf-22(ok693)* mutants. Previous studies have shown that these mutants tend to undergo LD fusion (37). We observed the process of two small LDs fusing into a larger LD in live imaging. Additionally, in another *daf-22(ok693)* mutant from the same experimental batch, we captured the interaction process between LDs and LROs, characterized by kiss-and-release dynamics (Fig. 4C and Supplemental Video 1 and 2). This mode of interaction between LROs and LDs resembles that observed in cells as reported in previous studies (38). Overall, BODIPY combined with FLF and Tau contrast imaging provides a convenient method for observing the dynamic interactions between LDs and between LDs and LROs.

## Discussion

BODIPY is a commercially available lipid dye known for its stability, ease of acquisition, and convenience in use. It has been effectively used for LD staining in various cell types (39, 40). However, its application in *C. elegans* has been less satisfactory due to off-target staining of LROs, leading to its gradual abandonment in *C. elegans* lipid research. Our study offers a potential turning point. The distinct chemical environments of LROs and LDs enable us to use fluorescence lifetime to distinguish between BODIPY-stained LROs and LDs.

By analyzing fluorescence lifetime distributions and applying appropriate lifetime gates, we were able to significantly reduce LRO fluorescence signals. Our proposed FLF method effectively minimizes the fluorescence interference from LROs on LDs, thereby improving the accuracy of lipid quantification. However, this enhancement comes at the expense of LD brightness. Our investigation into BODIPY concentration demonstrated that higher concentrations of BODIPY can further increase the RLL, partially compensating for the loss of LD visibility. Additionally, increasing excitation light intensity can help restore LD signal strength, ensuring it meets the requirements for proper observation.

Based on our findings, we identified that an 8 μM concentration of BODIPY, in combination with FLF, yields an optimal RLL while maintaining satisfactory LD visibility. For situations where high BODIPY concentrations are not feasible, increasing excitation light intensity still provides adequate LD visualization. Moreover, our results offer valuable insights for researchers without access to fluorescence lifetime imaging microscopes. Using 8 μM BODIPY alone modestly enhances the accuracy of fat storage quantification, even in the absence of advanced imaging techniques.

We foresee promising applications of these findings. First, the near-complete removal of BODIPY signals from LROs after 8 μM BODIPY staining and FLF allows the fluorescence intensity of BODIPY to accurately reflect fat storage, making this method suitable for fat storage quantification without fixation or freezing, making this method suitable for forward genetic screening. Our simulation experiments demonstrated potential high-throughput benefits. Additionally, combined with photo-highlighting, it anticipates being more suitable for large-scale screens (41). Moreover, by distinguishing LROs and LDs based on fluorescence lifetime, we can utilize the dual-target characteristic of BODIPY to observe the dynamic behaviors of LDs and LROs simultaneously. This simplifies the observation of LD-LD and LD-LRO interactions, which previously required specific expression of two fluorescent proteins. We anticipate that our research will facilitate the better application of BODIPY in future studies of worms’ LDs and LROs.

## Data availability

The data supporting this study are available in the article or available from the corresponding author upon reasonable request.

## Supplemental data

This article contains supplemental data.

## Conflict of interest

The authors declare that they have no conflicts of interest with the contents of this article.
