# Supplementary material for: A fluorescence lifetime-based novel method for accurate lipid quantification of BODIPY vital-stained C. elegans
Source: J Lipid Res. 2024 Sep 19;65(10):100646. doi: 10.1016/j.jlr.2024.100646 (PMC11530801; doi:10.1016/j.jlr.2024.100646)
Supplement: Supplemental Information [file mmc1.docx]

**SUPPLEMENTAL INFORMATION:**

# A Fluorescence Lifetime-Based Novel Method for Accurate Lipid Quantification of BODIPY Vital-Stained *C. elegans*

Chen Xu^1^, Jintao Luo^1^, Yong Yu^1*^

1. State Key Laboratory of Cellular Stress Biology, School of Life Sciences, Faculty of Medicine and Life Sciences, Xiamen University, Xiamen 361102, China

* Correspondence and requests for materials should be addressed to Yong Yu ([yuy@xmu.edu.cn](mailto:yuy@xmu.edu.cn))


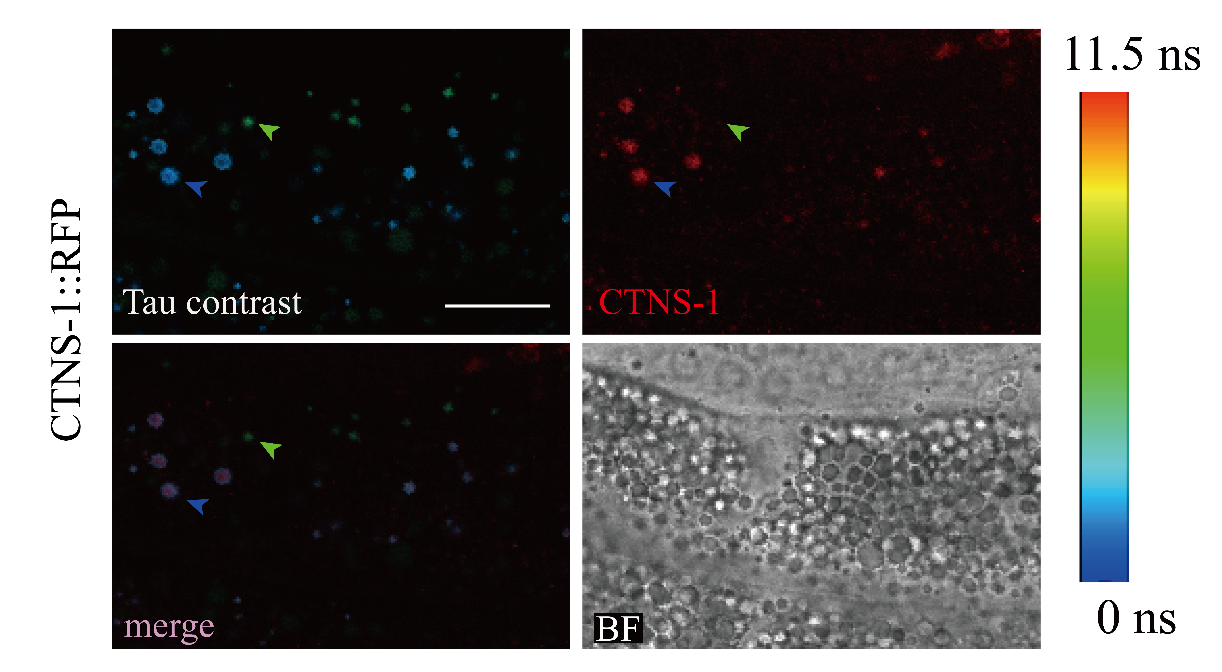


**Supplemental Fig. S1. LROs labeled with RFP co-locate with the blue region in Tau contrast image.**

CTNS-1::RFP transgenic worms were live-stained with 1 μM BODIPY. Lysosomes marked by red circles in the CTNS-1 channel overlapped with regions of short fluorescence lifetime (blue arrow) in Tau contrast images. The scale bar = 10 μm.


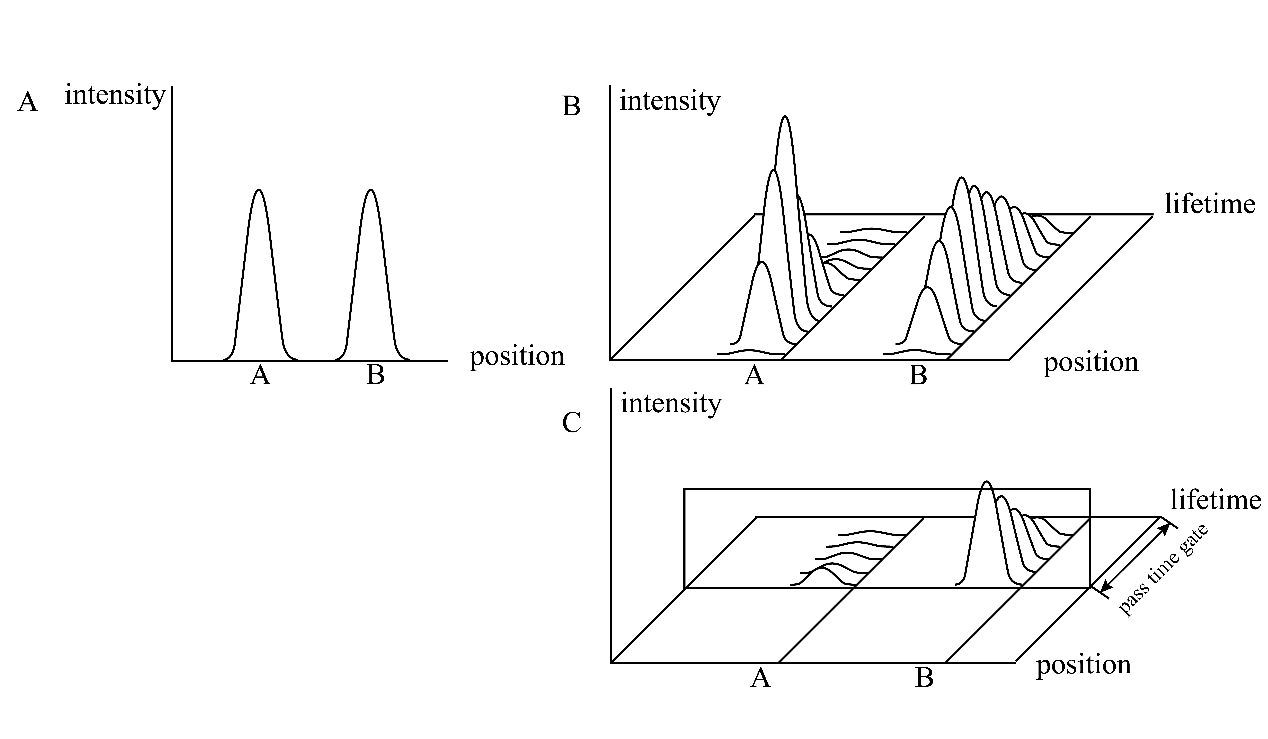


**Supplemental Fig. S2.** **Schematic illustration of the relationship among fluorescence lifetime distribution, fluorescence intensity, and FLF.**

(**A**) In normal confocal fluorescence images, it can’t be distinguished between two regions with identical fluorescence intensity. (**B**) In Tau Contrast images, two regions that possess identical fluorescence intensity exhibit different fluorescence lifetime distributions. (**C**) Based on fluorescence lifetime, a filter can be set to allow the collection of photons whose fluorescence lifetimes meet the time gate, thereby achieving fluorescence intensity filtering based on fluorescence lifetime.


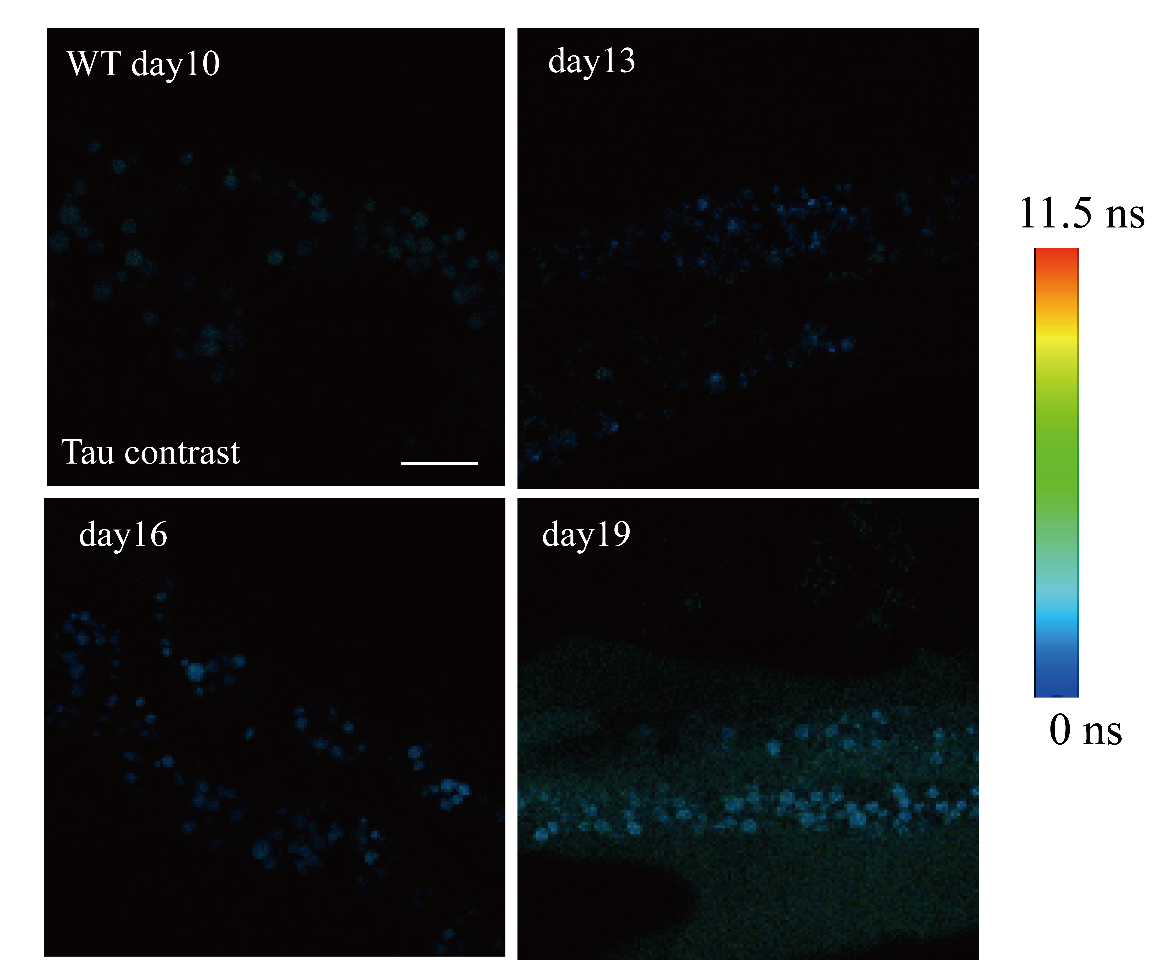


**Supplemental Fig. S3. Aging worms exhibit increased fluorescence intensity while maintaining a consistent short fluorescence lifetime, similar to LROs.**

Unstained worms were imaged using Tau contrast mode on different days of cultivation, revealing a continuously increasing spontaneous fluorescence in LROs, exhibiting short fluorescence lifetimes. The scale bar = 10 μm.

**Supplemental video 1. The process of two small LDs fusing into a larger LD.**

**Supplemental video 2. The interaction process between LDs and LROs.**
